# Synergistic Effect of Citric By-Product, Fibrolytic Enzyme and *Lactobacillus* spp. to Enhance Broiler Performance

**DOI:** 10.3390/ani15192815

**Published:** 2025-09-26

**Authors:** Nantanant Somparn, Padsakorn Pootthachaya, Warin Puangsap, Kittima Pattanasaeng, Chaiyapruek Hongladdaporn, Chanon Suntara, Anusorn Cherdthong, Perapong Phaengphairee, Sawitree Wongtangtintharn

**Affiliations:** 1 Department of Animal Science, Faculty of Agriculture, Khon Kaen University, Khon Kaen 40002, Thailand; nantanant.s@kkumail.com (N.S.); padsakornp@kkumail.com (P.P.); kittimapat@kkumail.com (K.P.); chanon_su@kkumail.com (C.S.); anusornc@kku.ac.th (A.C.); perpan@kku.ac.th (P.P.); 2 Department of Veterinary Public Health, Faculty of Veterinary Medicine, Khon Kaen University, Khon Kaen 40002, Thailand; warin.paun@kkumail.com; 3 Department of Animal Science Program, Faculty of Science and Technology, Loei Rajabhat University, Loei 42000, Thailand; chaiyapruek.h@gmail.com

**Keywords:** feed additives, growth performance, fibrolytic enzyme, *Lactobacillus* spp.

## Abstract

This study evaluated the effects of citric acid by-product (CABP), either alone or in combination with fibrolytic enzyme (F) and *Lactobacillus* spp. (L), in broiler diets. A total of 160 one-day-old male Arbor Acres broiler chicks (average initial body weight 43.0 ± 0.16 g) were randomly assigned to four CABP-based diets (4 replicates × 10 birds each): 12% CABP, CABP+F (0.05%), CABP+L (0.025%), and CABP+FL (0.05% F + 0.025% L). Birds were fed for 35 days in three phases: starter (days 1–10), grower (days 11–24), and finisher (days 25–35). Compared with the CABP-only group, broilers receiving CABP+F, CABP+L, or CABP+FL exhibited higher body weight and weight gain during the finisher and overall phases (*p* < 0.05). Carcass traits did not differ significantly among treatments, although meat hardness was reduced in the CABP+FL group (*p* < 0.05). Additive-supplemented groups also showed changes in intestinal morphology, with a lower villus height-to-crypt depth ratio compared with CABP alone. Red blood cell counts were not different between CABP and CABP+L, while hepatic enzyme activities (ALT, AST, ALP) remained unchanged across treatments, indicating no adverse effects on liver function. Overall, supplementation of CABP-based diets with fibrolytic enzymes and/or *Lactobacillus* spp. improved broiler performance and certain meat quality traits relative to CABP alone, without negative impacts on liver health or carcass yield. However, as no conventional corn–soy control diet was included, the results should be interpreted as comparisons within CABP-based diets.

## Simple Summary

This study evaluated the effects of using citric acid by-product (CABP) in combination with feed additives, namely fibrolytic enzymes and *Lactobacillus* spp., in broiler diets. CABP is considered a potential alternative energy source and a nutrient-rich ingredient for poultry. The evaluation covered growth performance, carcass quality, hematology, and economic return. Broilers fed CABP-based diets with enzyme and/or probiotic supplementation showed improved growth performance compared with CABP alone, without adverse effects on liver enzyme activities or carcass traits. Because all diets contained CABP and a conventional corn–soy control was not included, the findings should be interpreted as relative to CABP-only diets. Further studies are needed to confirm whether enzyme and probiotic supplementation can mitigate the limitations of CABP compared with standard commercial formulations.

## 1. Introduction

Citric acid is widely produced on an industrial scale through the process of aerobic fermentation using microorganisms such as *Aspergillus niger*. Under specific environmental conditions, citric acid is produced in excess as an overflow product along with a large amount of citric acid by-products [1]. In 2004, the global production of citric acid was reported to be about 1.4 million tonnes, with an annual growth rate of 3.5–4% to meet the rising demand for this acid [2,3]. Given the rising availability of citric acid by-products, exploring their value in livestock nutrition offers both economic and environmental benefits. According to Tanpong et al. [4], it has been reported that citric by-products account for approximately 70–80% of the total input of raw material. If not properly managed, the high volume of citric acid waste generated during production may raise environmental concerns. Improper disposal of these wastes into water sources, soil, or the atmosphere can lead to pollution and environmental degradation.

Citric acid by-products derived from industrial fermentation processes may retain nutritional components such as crude protein, fiber, and bioactive compounds (e.g., flavonoids and carotenoids) depending on the source and the processing method used [5]. Moreover, Tanpong et al. [6] reported that citric acid by-products contain 7.39% crude protein, 18.26% fiber, and a gross energy value of 3819 kcal/kg, supporting its potential as an alternative feed ingredient. Several studies have demonstrated that dietary supplementation with organic acid can enhance nutrient digestibility, improve intestinal morphology, and stimulate the proliferation of beneficial gut microbiota [7]. Furthermore, citric acid is an organic acid, and its by-products retain residual amounts that may confer similar beneficial effects [8,9]. However, Oryza et al. [10] and Tanpong et al. [11] reported that feeding poultry with 9–12% of untreated citric acid waste negatively affected their absorption of nutrients and reduced their growth performance, likely due to the acid’s high fiber content. This finding aligns with that of Jha and Mishra [12], who described dietary fiber as an antinutritional factor, as it can impair feed intake and nutrient digestibility. The antinutritive effects of fiber include the formation of viscous intestinal contents, which slow down feed passage, impair nutrient accessibility to digestive enzymes, and decrease overall nutrient digestibility. These factors can lead to reduced feed intake, poor feed conversion efficiency, and compromised growth performance in broiler chickens, ultimately resulting in significant economic losses for commercial poultry producers. To address these challenges, the poultry industry has actively explored alternative feed ingredients and innovative feed additives. Notably, recent advances in enzyme technology and probiotic supplementation have been shown to be promising in mitigating the antinutritive effects of dietary fiber by enhancing fiber degradation, improving gut health, and optimizing nutrient utilization efficiency in fiber-rich diets. To address the limitations associated with fiber in citric waste, the inclusion of enzymes has been explored as a strategy to improve its utilization in poultry diets. Fibrolytic enzymes have gained particular attention. These enzymes, categorized as exogenous enzymes, are capable of breaking down non-starch polysaccharides such as cellulose. They function by cleaving β-1,4-glycosidic bonds in cellulose, releasing glucose as a product [13]. This enzymatic action also enhances the availability of digestible energy for animals [14].

In addition to enzyme supplementation, probiotics have also been studied as an effective approach to enhance the utilization of high-fiber feed ingredients [15,16]. To improve the quality of citric acid by-products, Tanpong et al. [4] investigated the nutritional enrichment of citric acid residue through fermentation with *Bacillus subtilis* I9. They conducted fermentation for 72 and 96 h, and this resulted in a significantly improved chemical composition, increasing the crude protein (CP) content to 9.01% and reducing the crude fiber (CF) content to 16.28%. Similarly, previous research has demonstrated that the use of *Bacillus amyloliquefaciens* and humic substances enhances the content of protein and fiber in fermented rice bran [14]. Moreover, another study using a 1:1 ratio *of Lactobacillus fermentum* and *Bacillus subtilis* (solid-state fermented rapeseed meal) also showed improvements in terms of the nutrient composition of feed ingredients [17]. Among various probiotic strains, *Lactobacillus* spp. have been widely used due to their acid tolerance, ability to colonize the gut, and contribution to enzymatic digestion [18,19]. These beneficial bacteria can improve intestinal health, reduce pathogenic bacterial loads, and stimulate immune responses in poultry [20,21]. The combined application of probiotics and fibrolytic enzymes presents a promising strategy to overcome the limitations associated with high-fiber by-products [22,23]. While enzymes degrade complex fibers into simpler, absorbable forms Suntara et al. [13], probiotics support the microbial balance and fermentation capacity in the gut, leading to synergistic improvements in nutrient utilization and overall performance.

Therefore, evaluating the use of citric acid by-products (CABP) as a sustainable feed ingredient in broiler production is of considerable importance. The present study investigated the effects of incorporating 12% CABP, either alone or supplemented with fibrolytic enzyme and *Lactobacillus* spp., on growth performance, hematological parameters, small intestinal morphology, carcass traits, and meat quality in broilers. Since all experimental diets contained CABP, the outcomes reflect comparisons among CABP-based diets rather than against a conventional corn–soy control. Nevertheless, the study provides valuable insights into how enzyme and probiotic supplementation may improve the utilization of CABP, thereby supporting its potential role as an alternative feed ingredient to reduce reliance on conventional feed resources.

## 2. Materials and Methods

### 2.1. Animal Ethics

This study was conducted according to the guidelines for animal experimentation established by the National Research Council of Thailand. The research protocol was reviewed and approved by the Institutional Animal Care and Use Committee (IACUC) of Khon Kaen University, Khon Kaen, Thailand (Approval No. IACUC-KKU-47/67). All experimental procedures were conducted at the Poultry Research Farm, Faculty of Agriculture, Khon Kaen University.

### 2.2. Birds, Housing, and Experimental Diet

A total of 160 one-day-old male Arbor Acres broiler chicks (average initial body weight of 43.0 ± 0.16 g) were obtained from a commercial poultry hatchery (Charoen Pokphand Group Co., Ltd., Nakhon Ratchasima, Thailand). The chicks were vaccinated against Infectious Bronchitis and Newcastle disease virus and reared in an open housing system at the Poultry Research Farm, Faculty of Agriculture, Khon Kaen University, Thailand. The birds were randomly assigned to four dietary treatments in a 35-day feeding trial, with four replicates per treatment and 10 birds per replicate. The experimental period was divided into three feeding phases: starter (1–10 days), grower (11–24 days), and finisher (25–35 days). The dietary treatments were as follows: (CABP): Replacing corn with 12% citric acid by-product; (CABP+F): CABP diet supplemented with 0.05% fibrolytic enzyme per 100 kg of feed. (CABP+L): CABP diet supplemented with 0.025% probiotic (*Lactobacillus* spp. 1.0 × 10^11^ CFU/kg); (CABP+FL): CABP diet supplemented with 0.05% fibrolytic enzyme and 0.025% probiotic (*Lactobacillus* spp. 1.0 × 10^11^ CFU/kg). It should be noted that all diets contained 12% CABP. A conventional corn–soy control diet without CABP was not included; therefore, the experimental comparisons are restricted to CABP-based diets. The CABP (11.68% CP, 9.82% moisture, 18.46% crude fiber, 1417 kcal/kg) was sourced from Chok Phalitsap Co., Ltd., Chachoengsao, Thailand; fibrolytic enzyme (Digegrain-Delta^®^) from Union Castap Co., Ltd., Bangkok, Thailand; and *Lactobacillus* spp. from Innovet Corporation Co., Ltd., Samut Prakan, Thailand. The nutritional formulation of the experimental diet is provided in Table 1.

### 2.3. Sample Collection and Measurement

#### 2.3.1. Growth Performance and Economic Return

The performance parameters were recorded for each pen of the study: Body weight (BW) and feed intake (FI) were recorded on days 10, 24, and 35 days of age to calculate body weight gain (BWG) and feed conversion ratio (FCR). The survival rate (SR) was also recorded throughout the experimental period. Economic returns were calculated as feed cost per gain (FCG), sale per bird revenue (SBR), net profit per bird (NPR), and return on investment (ROI).

$$FCG=\frac{FI\left(kg\right)\times Feed\;Cost\;per\;kg}{Total\;BWG\;(kg)}$$

$$SBR=\left(\frac{NPR}{COST}\right)\times 100$$

$$NPR=\left(Total\;live\;weight\;sold\times Market\;price/kg\right)-(Feed\;cost+Chick\;cost)$$

$$ROI=\left(\frac{NPR}{COST}\right)\times 100$$

#### 2.3.2. Carcass Traits and Meat Qualities

At the end of the trial (35 days of age), two birds per replicate were randomly selected for carcass trait evaluation. Birds were fasted for 12 h before slaughter. Live body weight was recorded before euthanasia by cervical dislocation. Carcasses were eviscerated and processed according to Diarra et al. [24], then dissected and weighed by individual part. Dressing percentage and relative organ weights were calculated. Carcass parts included breast fillet, inner breast fillet, whole wing, thigh, and drumstick.

Afterward, the breast samples were collected for quality analysis, including color, pH, drip loss, cooking loss, shear force, and texture profile analysis (TPA). The pH of meat samples was measured using a pH meter (HI98163, Hanna Instruments, Villafranca Padovana, Italy) with a FC2323 penetration probe, calibrated with pH 4.01, 7.01, and 10.01 buffers prior to use. Drip loss was measured by weighing raw meat before and after 24 h storage at 4 °C in a suspended vertical position. Cooking loss was determined after heating samples in a water bath at 85 °C until reaching an internal temperature of 80 °C, followed by cooling to 4 °C and reweighing.

The TPA was conducted on cooked samples cut into 1 × 1 × 1 cm cubes to assess hardness, cohesiveness, and chewiness. Shear force was evaluated using 1 × 2 × 1 cm pieces. Texture was analyzed using a TA.HDplusC texture analyzer (Stable Micro Systems, Godalming, UK). A 50 mm cylindrical aluminum probe was used for TPA, and a V-slot Warner–Bratzler blade was used for shear force. Data was recorded via Exponent software (version 6.2, Stable Micro Systems, Godalming, UK). The method followed and calculated following description of [25].

#### 2.3.3. Analysis of Hematology Profiles

Blood samples were collected at 35 days of age following the method described by Somparn et al. [25]. A 5 mL blood sample was collected from the wing vein of each bird using sterile syringes and subsequently divided into two portions based on the type of analysis required. The first portion, consisting of 4 mL of blood without anticoagulants (ethylenediaminetetraacetic, EDTA) acid, was centrifuged at 3000 rpm for 10 min at 4 °C to separate the serum. This serum was analyzed for hepatic enzyme activities, including alanine aminotransferase (ALT), aspartate aminotransferase (AST), and alkaline phosphatase (ALP), using an auto-chemistry analyzer (Chiron, Emeryville, CA, USA).

The second portion, comprising 1 mL of blood containing EDTA as an anticoagulant, was used for hematological analysis. Red blood cell (RBC), white blood cell (WBC), hematocrit, hemoglobin, mean corpuscular volume (MCV), mean corpuscular hemoglobin (MCH), and mean corpuscular hemoglobin concentration (MCHC) were determined using an automated hematology analyzer (Sysmex XE-2100, Kobe, Japan). Hematology analysis was performed at Vet Central Lab Co., Ltd., Khon Kaen, Thailand.

#### 2.3.4. Small Intestine Morphology

At 24 and 35 days, two birds per treatment were randomly selected and slaughtered for intestinal sample collection, specifically the middle segments of the duodenum, jejunum, and ileum. Method described by De Verdal et al. [26], approximately 0.5 cm of each intestinal section was excised and preserved in 10% neutral-buffered formalin at room temperature for subsequent histological analysis. Nevertheless, the intestinal sample was rinsed with phosphate-buffered saline (PBS) and kept at 4 °C prior to further processing. For tissue sectioning, the samples were embedded in an appropriate cryo-embedding medium (Cryomatrix^®^, 4481 Campus Drive, Kalamazoo, MI, USA) and rapidly frozen using liquid nitrogen. Cross-sections with a thickness of 10 μm were prepared at −20 °C using a cryostat and mounted onto gelatin-coated microscope slides for histological analysis. Subsequently, intestinal morphology was assessed using a Leica microscope (40× magnification; Aperio CS2 Leica microscope, Vista, CA, USA), and the villus height (VH) and crypt depth (CD) were measured using SlideViewer software (version 2.7). The villus height-to-crypt depth ratio (V/C ratio) was calculated following the method described by [26].

### 2.4. Statistical Analysis

The experiment was conducted using a completely randomized design. Data was analyzed using one-way analysis of variance (ANOVA) to evaluate the effects of dietary treatments. When significant differences were detected among means with a *p*-value of less than 0.05. Duncan’s multiple range test and Orthogonal contrast were used for treatment comparisons. All statistical analyses were performed using SAS version 9.1 software (Statistical Analysis System, SAS Institute Inc., Cary, NC, USA). The statistical model was as follows:

Y_ijk_ = μ + α_i_ + ∑_ij_,

when Y_ijk_ = observed variable; μ = Overall mean; α_i_ = Treatments (CABP, CABP+F, CABP+L, and CABP+FL); ∑_ij_ = Experiment error.

## 3. Results 

### 3.1. Growth Performance

The results of growth performance on broilers are shown in Table 2. During the starter phases, there were no statistically significant differences (*p* > 0.05) observed in any of the measured parameters. However, the mean values indicated a trend toward improved FI and FCR in groups supplemented with feed additives. Additionally, in the grower and finisher phases, the groups receiving dietary supplements (CABP+F, CABP+L, and CABP+FL) exhibited significantly better FCR values (*p* < 0.05) compared to the CABP-based diets, particularly in the groups supplemented with single additives (CABP+F and CABP+L). Furthermore, during the overall phase, BW and BWG were significantly higher (*p* < 0.05) in the supplemented groups than CABP group. Although no significant differences were observed in FCR during the overall phase, a positive trend was noted in the feed additive groups, indicating a potential improvement in feed efficiency.

### 3.2. Economic Returns

The results of the economic return analysis (Table 3) indicated that the costs in the CABP group throughout all experimental phases were significantly lower than in the other groups (*p* < 0.0001). No significant differences were observed for the other parameters. However, the overall results demonstrated that SBR and NPR in the CABP+F, CABP+L, and CABP+FL groups were significantly higher than in the CABP group (*p* < 0.05).

### 3.3. Carcass Yield and Meat Quality

The carcass traits are presented in Table 4. As indicated that dietary supplementation with CABP+L and CABP+FL significantly increased heart percentage compared to the other groups, although no significant difference was observed when compared with the CABP group (*p* < 0.05). However, no other significant differences were found in external organ weights among the experimental groups.

The meat quality parameters are presented in Table 5. At 24 h, pH values differed significantly among treatments (*p* < 0.05), supplemented groups with feed additives showed lower pH values than the CABP group, particularly in the CABP+FL group. In terms of meat hardness was considerably lower in the CABP+FL group (*p* < 0.05), indicating improved hardness. No significant differences were observed in meat color, drip loss, cooking loss, cohesiveness, chewiness, or shear force

### 3.4. Hematology Profiles

The effects of the dietary treatments on hematological profiles at 35 days of age are shown in Table 6. Results showed that the CABP group showed a significant improvement in RBC compared to the other groups (*p* < 0.05), although no significant difference was observed between the CABP and CABP+L groups. WBC counts did not differ significantly among treatments (*p* > 0.05). However, MCV values differed significantly among the treatment groups (*p* = 0.03). The groups receiving feed additives exhibited significantly higher MCV values compared to the CABP group. However, no significant difference was observed between the CABP+F and CABP groups. Moreover, the CABP+FL group showed a significantly higher MCH than other groups (*p* < 0.05). Regarding liver enzyme activities (i.e., ALT, AST, ALK), no significant differences were found among treatments (*p* < 0.05).

### 3.5. Small Intestinal Morphology

The effects of diet experimentally on intestinal morphology in broilers at 24 and 35 day of age are shown in Table 7. In the duodenum and ileum of 24 and 35 day, there was no difference in the villus height, crypt depth, and V/C among treatments (*p* > 0.05). However, in the jejunum at 24 days of age, the V/C ratio was highest in the CABP group (*p* < 0.05).

## 4. Discussion 

The present study indicated that the inclusion of CABP in broiler diets, particularly when combined with fibrolytic enzymes and *Lactobacillus* spp., was associated with improved growth performance during the finisher phase (25–35 days). In contrast, no significant differences were observed during the starter and grower phases, which may reflect the underdeveloped digestive and microbial systems of younger broilers [27]. This interpretation is in line with the findings of Adil et al. [28], who reported that organic acid supplementation had limited effects during early broiler growth due to immature digestive and immune systems. During the finisher phase, birds receiving CABP+F, CABP+L, or CABP+FL diets showed higher BW and BWG, along with a numerical improvement in FCR. These patterns agree with Tejeda and Kim [29], who observed enhanced growth performance with rice-derived CABP at optimal inclusion levels. Similarly, Chowdhury et al. [30] and Boling et al. [31] suggested that growth-promoting effects of CABP may be partly related to improved utilization of minerals such as phosphorus and calcium, which are essential for skeletal development and nutrient absorption. The potential synergistic interaction between fibrolytic enzymes and probiotics may also have contributed to the observed responses. Fibrolytic enzymes can reduce the impact of anti-nutritional factors (ANFs) and enhance nutrient availability by breaking down complex fiber structures [32], while *Lactobacillus* spp. help to maintain a beneficial microbial balance and reduce pathogenic bacteria [33,34]. Together, these mechanisms may support nutrient uptake and overall performance [20]. Although FCR differences were not statistically significant across all phases, the downward trend observed in the CABP+FL group suggests possible improvements in feed efficiency. The ability of CABP combined with probiotics and enzymes to improve broiler growth highlights its potential as a sustainable feed alternative, reducing reliance on conventional corn–soy diets. Comparable outcomes were reported by Lutful Kabir [18], who found that citric acid supplementation improved FCR and BWG when combined with microbial or enzymatic additives.

Carcass characteristics were not markedly altered by the treatments, with no significant differences in breast, thigh, or wing percentages. The heart percentage was, however, higher in the CABP+FL group than in CABP+F, which may indicate elevated circulatory activity or metabolic demand associated with greater growth [35]. Abdel-Fattah et al. [36] also reported that organic acid supplementation could stimulate the development of metabolically active organs, including the heart, through improved nutrient absorption and blood circulation. The absence of significant changes in liver, spleen, or abdominal fat weights further supports the lack of obvious toxicity of CABP under the conditions of this study.

Meat quality responses showed that 24 h postmortem pH values differed among treatments, with the CABP-only group having the highest pH. An elevated pH has been linked to improved water-holding capacity and tenderness due to reduced protein denaturation during postmortem glycolysis [37]. Additionally, meat hardness was lower in the CABP+FL group, indicating better tenderness. While shear force is a standard measure of tenderness, texture profile analysis provides complementary information, and the reduction in hardness can be considered an indicator of improved textural quality. These effects may relate to the combined action of enzymes and probiotics on fiber degradation, amino acid availability, protein metabolism, gut microbial balance, and postmortem proteolysis, which together could influence meat texture and shelf-life [38].

In terms of hematological profiles, CABP inclusion influenced RBC, MCV, and MCH values, while other blood parameters and liver enzyme activities (ALT, AST, ALK) were unaffected. These changes in red cell indices may suggest enhanced erythropoiesis and oxygen-carrying capacity, thereby supporting physiological function. Reena et al. [39] reported that CABP fermented from cassava with *Aspergillus niger* retains residual citric acid, which may contribute to such effects. Chowdhury et al. [30] and Samanya and Yamauchi [40] also found that organic acid supplementation improved hematological traits in broilers, likely through enhanced nutrient absorption and reduced gut pathogen loads. The absence of changes in serum liver enzyme activities suggests that CABP did not induce hepatotoxic effects under the present conditions. This interpretation is consistent with El-Sanhoury and Ahmed [41], who reported no adverse impacts of multi-enzyme supplementation on ALT, AST, or liver histology in broilers, and with Gong et al. [42], who observed that Bacillus probiotics improved antioxidant capacity without altering liver enzyme activities.

The intestinal morphology analysis indicated that at 24 days, CABP-fed birds tended to show a higher villus height to crypt depth (V/C) ratio, suggesting a possible improvement in absorptive surface area. This observation corresponds with Oryza et al. [43], who reported favorable effects of CABP on intestinal morphology and microbial balance. In the CABP+L group, a higher V/C ratio was also observed, which may reflect a combined effect of probiotics and residual organic acids in CABP. A similar trend was described by Rodjan et al. [44], who found that *Lactobacillus* spp. with organic acids enhanced intestinal development and nutrient utilization.

Taken together, these findings suggest that enzyme and probiotic supplementation can enhance the use of CABP in broiler diets. However, because all experimental treatments contained 12% CABP and no conventional corn–soy control diet was included, the observed effects should be interpreted as improvements relative to CABP-only diets. Future work should include conventional controls to determine whether enzyme and probiotic supplementation can fully counterbalance the limitations of CABP compared with standard commercial formulations.

## 5. Conclusions

This study indicates that supplementing citric acid by-product (CABP) diets with fibrolytic enzymes and/or *Lactobacillus* spp. improved broiler performance during the finisher and overall phases, as evidenced by higher body weight and weight gain. Positive effects were also observed on red blood cell indices and meat tenderness, while liver enzyme activities and carcass traits were not adversely affected. These outcomes suggest that enzyme and probiotic supplementation can enhance the utilization of CABP in broiler diets, thereby supporting its potential as a sustainable feed ingredient. Importantly, because all experimental treatments contained 12% CABP and a conventional corn–soy control diet was not included, the present findings should be interpreted as improvements relative to CABP-only diets. Future studies incorporating conventional control diets are needed to determine whether such supplementation strategies can offset the limitations of CABP when compared with standard commercial formulations.

## Figures and Tables

**Table 1:** Ingredients formula of the experimental diet used in the study (as dry basis).

Ingredients	Starter 1–10 d				Grower 11–24 d				Finisher 25–35 d			
	CABP	CABP+F	CABP+L	CABP+FL	CABP	CABP+F	CABP+L	CABP+FL	CABP	CABP+F	CABP+L	CABP+FL
Corn	36.70	36.70	36.70	36.70	38.20	38.20	38.20	38.20	45.20	45.20	45.20	45.20
Soybean meal 45%	26.65	26.65	26.65	26.65	23.90	23.90	23.90	23.90	18.00	18.00	18.00	18.00
^1^ CABP	12.00	12.00	12.00	12.00	12.00	12.00	12.00	12.00	12.00	12.00	12.00	12.00
Palm oil	2.00	2.00	2.00	2.00	2.40	2.40	2.40	2.40	2.30	2.30	2.30	2.30
Full-fat SBM	18.00	18.00	18.00	18.00	19.00	19.00	19.00	19.00	18.00	18.00	18.00	18.00
Choline chloride	0.10	0.10	0.10	0.10	0.10	0.10	0.10	0.10	0.10	0.10	0.10	0.10
limestone	1.65	1.65	1.65	1.65	1.60	1.60	1.60	1.60	1.60	1.60	1.60	1.60
Dicalcium phosphate P21%	1.80	1.80	1.80	1.80	1.70	1.70	1.70	1.70	1.70	1.70	1.70	1.70
Salt	0.40	0.40	0.40	0.40	0.40	0.40	0.40	0.40	0.40	0.40	0.40	0.40
DL-Methionine	0.30	0.30	0.30	0.30	0.30	0.30	0.30	0.30	0.30	0.30	0.30	0.30
L-Lysine	0.15	0.15	0.15	0.15	0.15	0.15	0.15	0.15	0.15	0.15	0.15	0.15
^2^ Premix	0.25	0.25	0.25	0.25	0.25	0.25	0.25	0.25	0.25	0.25	0.25	0.25
Total	100.00	100.00	100.00	100.00	100.00	100.00	100.00	100.00	100.00	100.00	100.00	100.00
Fibrolytic enzyme	0.00	0.00	0.00	0.05	0.00	0.05	0.00	0.05	0.00	0.05	0.00	0.05
*Lactobacillus* spp.	0.00	0.00	0.025	0.025	0.00	0.00	0.025	0.025	0.00	0.00	0.025	0.025
Nutrient composition by calculated												
Crude protein, %	22.51	22.53	22.53	22.53	21.63	21.76	21.76	21.76	19.15	19.30	19.30	19.30
Crude fiber, %	5.50	5.50	5.50	5.50	5.43	5.43	5.43	5.43	5.08	5.08	5.08	5.08
Metabolizable energy, kcal/kg	3000	3000	3000	3000	3082	3082	3082	3082	3130	3130	3130	3130

^1^ CABP: Citric acid by-product; ^2^ Provided per kilogram of diet: 4.80 MIU of vitamin A, 2.00 MIU of vitamin D3, 30,000 IU of vitamin E, 1.20 g of vitamin K3, 1.20 g of vitamin B1, 3.20 g of vitamin B2, 2.00 g of vitamin B6, 0.0064 g of vitamin B12, 24.00 g of niacin, 0.80 g of folic acid, 0.08 g of biotin, and 6.00 g of pantothenic acid. 40.00 g of Zn, 48.00 g of Mn, 16.00 g of Fe, 6.40 g of Cu, 0.50 g of I, 0.04 g of Co, and 0.12 g of Se. 0.20 g of antioxidant, 0.88 g of anticaking agent, and 1.00 kg of carrier.

**Table 2:** Effects of dietary treatments on growth performance parameters.

Items	Treatments				SEM	*p*-Value	Orthogonal Contrast		
	CABP	CABP+F	CABP+L	CABP+FL			Contrast 1	Contrast 2	Contrast 3
Initial BW, g	43.08	43.18	42.70	43.00	2.13	0.96	0.839	0.478	0.934
Starter, 1–10 d									
BW, g	220.10	218.20	220.13	211.33	1.49	0.47	0.152	0.065	0.369
BWG, g	177.02	175.03	176.93	168.33	1.50	0.50	0.196	0.096	0.410
FI, g	265.70	244.93	246.33	243.13	2.18	0.34	0.004	0.053	0.501
FCR	1.51	1.40	1.39	1.45	0.16	0.38	<0.0001	0.356	0.003
SR	100.00	100.00	100.00	100.00	N/A	N/A	N/A	N/A	N/A
PI	118.78	125.23	127.25	117.04	1.67	0.52	0.899	0.201	0.801
Grower, 11–24 d									
BW, g	940.70	964.93	958.37	968.68	3.28	0.80	0.061	0.048	0.069
BWG, g	720.60	746.72	738.25	757.35	3.05	0.57	0.095	0.065	0.191
FI, g	1912.23	1615.05	1745.03	1618.65	6.83	0.70	0.252	0.778	0.343
FCR	2.24 ^a^	2.17 ^a^	2.35 ^a^	2.14 ^b^	0.10	0.02	0.007	0.008	0.146
SR	100.00	100.00	100.00	100.00	N/A	N/A	N/A	N/A	N/A
PI	231.05	248.39	225.35	254.09	2.56	0.39	0.446	0.303	0.237
Finisher, 25–35 d									
BW, g	1760.33 ^b^	1896.73 ^a^	1917.00 ^a^	1924.18 ^a^	10.89	0.03	0.009	0.929	0.919
BWG, g	819.63 ^b^	931.80 ^a^	958.63 ^a^	955.51 ^a^	3.64	0.009	0.035	0.628	0.372
FI, g	1507.68	1541.43	1540.00	1633.96	5.15	0.41	0.270	0.263	0.757
FCR	1.84 ^a^	1.65 ^b^	1.61 ^b^	1.72 ^b^	0.10	0.02	0.025	0.132	0.412
SR	100.00	100.00	100.00	97.50	0.790	0.43	0.205	0.128	1.000
PI	405.96	513.13	546.77	500.41	4.10	0.06	0.056	0.487	0.493
Overall, 1–35 d									
BW, g	1760.33 ^b^	1896.73 ^a^	1917.00 ^a^	1924.18 ^a^	4.32	0.03	0.004	0.929	0.919
BWG, g	1717.25 ^b^	1853.55 ^a^	1873.80 ^a^	1881.18 ^a^	4.33	0.03	0.004	0.930	0.929
FI, g	3385.60	3401.40	3531.35	3495.73	8.12	0.83	0.894	0.446	0.575
FCR	1.97	1.84	1.88	1.86	0.16	0.36	0.028	0.508	0.735
SR	100.00	100.00	100.00	97.50	0.790	0.43	0.574	0.128	1.000
PI	249.03	288.83	285.88	283.37	2.44	0.12	0.012	0.790	0.864

BW: Body weight; BWG: Body weight gain; FI: Feed intake; FCR: Feed conversion ratio; SR: Survival rate; PI: Productivity index; N/A: Not applicable; ^a,b^ Means within a row with different superscripts are different (*p* < 0.05); SEM: Standard error of mean; Contrast 1: CABP vs. CABP+F, CABP+L, CABP+FL; Contrast 2: CABP+F, CABP+L vs. CABP+FL; Contrast 3: CABP+F vs. CABP+L.

**Table 3:** The effects of dietary supplementation with citric acid by-product on economic returns.

Items	Treatments				SEM	*p*-Value	Orthogonal Contrast		
	CABP	CABP+F	CABP+L	CABP+FL			Contrast 1	Contrast 2	Contrast 3
Starter, 1–10 d									
Cost, (baht)	18.51 ^d^	18.60 ^b^	18.52 ^c^	18.61 ^a^	N/A	<0.0001	<0.0001	<0.0001	<0.0001
Feed cost, (baht/bird)	4.92	4.56	4.56	4.52	0.30	0.38	0.161	0.878	0.977
FCG, (baht/bird)	27.85	26.01	25.76	26.92	0.68	0.41	0.482	0.382	0.851
Grower, 11–24 d									
Cost, (baht)	18.53 ^b^	18.62 ^a^	18.53 ^b^	18.62 ^a^	N/A	<0.0001	<0.0001	<0.0001	<0.0001
Feed cost, (baht/bird)	29.87	30.01	32.34	30.14	0.93	0.72	0.670	0.625	0.374
FCG, (baht/bird)	41.53	40.40	43.53	39.85	0.98	0.56	0.828	0.383	0.268
Finisher, 25–35 d									
Cost, (baht)	17.84 ^d^	17.93 ^b^	17.85 ^c^	17.94 ^a^	N/A	<0.0001	<0.0001	<0.0001	<0.0001
Feed cost, (baht/bird)	26.90	27.64	27.49	29.31	0.69	0.35	0.120	0.158	0.914
FCG, (baht/bird)	32.84	29.64	28.74	31.76	0.85	0.22	0.532	0.170	0.664
Overall, 1–35 d									
Cost, (baht)	18.29 ^d^	18.38 ^b^	18.30 ^c^	18.39 ^a^	N/A	<0.0001	<0.0001	<0.0001	<0.0001
Feed cost, (baht/bird)	61.93	62.53	64.62	64.29	1.10	0.83	0.513	0.814	0.550
FCG, (baht/bird)	36.05	33.74	34.41	35.22	0.78	0.58	0.277	0.457	0.705
SBR	128.79 ^b^	139.02 ^a^	140.54 ^a^	141.09 ^a^	1.19	0.03	0.004	0.709	0.709
NPR	92.74 ^b^	105.27 ^a^	106.13 ^a^	105.87 ^a^	1.25	0.03	0.004	0.966	0.850
ROI	257.34	312.42	310.06	304.49	2.92	0.13	0.022	0.752	0.924

Cost: total feed cost; Feed cost: total feed per bird; FCG: feed cost per gain; SBR: sale per bird revenue; NPR: net profit per bird; ROI: return on investment; N/A: Not applicable; ^a–d^ Means within a row with different superscripts are different (*p* < 0.05); SEM: Standard error of means; Contrast 1: CABP vs. CABP+F, CABP+L, CABP+FL; Contrast 2: CABP+F, CABP+L vs. CABP+FL; Contrast 3: CABP+F vs. CABP+L.

**Table 4:** The effects of dietary supplementation with citric acid by-product on broiler slaughter weight and carcass, when 35 d.

Items	Treatments				SEM	*p*-Value	Orthogonal Contrast		
	CABP	CABP+F	CABP+L	CABP+FL			Contrast 1	Contrast 2	Contrast 3
Dressing percentage, %	80.74	78.75	79.91	83.32	0.90	0.28	0.965	0.066	0.620
Internal organ, %									
Liver	2.42	2.32	2.29	2.39	0.26	0.90	0.611	0.603	0.909
Heart	0.52 ^ab^	0.46 ^b^	0.52 ^ab^	0.57 ^a^	0.10	0.03	0.822	0.009	0.089
Pancreas	0.30	0.27	0.29	0.30	0.08	0.42	0.459	0.301	0.283
Spleen	0.14	0.20	0.15	0.19	0.11	0.33	0.252	0.757	0.157
Gizzard	1.54	1.43	1.52	1.51	0.16	0.49	0.374	0.628	0.246
Abdominal fat	1.39	2.49	1.10	1.72	0.62	0.62	0.672	0.942	0.222
External organ, %									
Wing	11.00	10.95	10.87	11.06	0.35	0.95	0.874	0.624	0.816
Thigh	17.89	18.97	17.83	18.76	0.51	0.33	0.281	0.424	0.437
Drumstick	14.59	14.28	13.95	14.41	0.37	0.48	0.321	0.586	0.149
Breast inner fillet	5.79	5.84	5.71	5.65	0.22	0.57	0.657	0.329	0.354
Breast fillet	27.97	29.27	28.31	28.13	0.48	0.25	0.286	0.267	0.170

^a,b^ Means within a row with different superscripts are different (*p* < 0.05); SEM: Standard error of means; Contrast 1: CABP vs. CABP+F, CABP+L, CABP+FL; Contrast 2: CABP+F, CABP+L vs. CABP+FL; Contrast 3: CABP+F vs. CABP+L.

**Table 5:** The effects of dietary supplementation with citric acid by-product on the meat quality of broiler, when 35 d.

Items	Treatments				SEM	*p*-Value	Orthogonal Contrast		
	CABP	CABP+F	CABP+L	CABP+FL			Contrast 1	Contrast 2	Contrast 3
pH									
0 h	6.23	6.11	6.19	6.26	0.19	0.60	0.636	0.264	0.456
24 h	6.25 ^a^	6.05 ^b^	6.04 ^b^	5.97 ^b^	0.17	0.02	0.004	0.271	0.876
Meat color									
L*	48.10	48.19	49.85	50.85	0.76	0.30	0.269	0.217	0.325
a*	−0.72	−0.98	−0.70	−0.84	0.37	0.87	0.705	0.991	0.471
b*	0.33	0.13	0.53	0.85	0.54	0.54	0.240	0.486	0.640
Meat physical									
Drip loss, %	3.98	4.29	2.69	4.51	0.55	0.19	0.828	0.187	0.082
Cooking loss, %	14.22	16.65	12.92	13.05	0.98	0.52	0.994	0.475	0.197
Shear force, g/cm^2^	2006.86	2923.52	2772.15	2366.05	14.63	0.45	0.194	0.377	0.807
Hardness, g	920.02 ^a^	895.31 ^a^	856.31 ^a^	638.62 ^b^	5.57	0.03	0.111	0.009	0.664
Cohesiveness	0.60	0.60	0.62	0.64	0.09	0.32	0.373	0.148	0.426
Chewiness	359.91	394.29	323.18	215.89	4.36	0.10	0.086	0.067	0.601

L* = lightness; a* = redness; b* = yellowness; ^a,b^ Means within a row with different superscripts are different (*p* < 0.05); SEM: Standard error of means; Contrast 1: CABP vs. CABP+F, CABP+L, CABP+FL; Contrast 2: CABP+F, CABP+L vs. CABP+FL; Contrast 3: CABP+F vs. CABP+L.

**Table 6:** Hematology profile of dietary supplementation with citric acid by-product (CABP), when 35 d.

Items	Treatments				SEM	*p*-Value	Orthogonal Contrast		
	CABP	CABP+F	CABP+L	CABP+FL			Contrast 1	Contrast 2	Contrast 3
Hematology profiles									
RBC, ×10^6^ cells/mm^3^	2.66 ^a^	2.34 ^b^	2.46 ^ab^	2.30 ^b^	0.19	0.02	0.006	0.321	0.258
Hemoglobin, g/dL	17.70	15.88	16.93	16.50	0.53	0.19	0.076	0.887	0.213
Hematocrit, %	30.25	28.50	30.75	29.00	0.59	0.12	0.313	0.470	0.039
WBCs, cells/μL	9790	9020	8140	8938	27.54	0.90	0.545	0.851	0.689
Heterophils, %	12.75	14.75	14.75	12.50	1.06	0.82	0.637	0.427	1.000
Basophil, %	14.00	12.50	11.25	9.5	0.96	0.40	0.195	0.313	0.970
Lymphocytes, %	72.25	71.75	73.00	77.00	1.35	0.73	0.698	0.318	0.817
Monocytes, %	1.00	1.00	1.00	1.00	N/A	N/A	N/A	N/A	N/A
H/L ratio	0.19	0.21	0.20	0.17	0.14	0.88	0.901	0.441	0.896
MCV	113.65 ^b^	122.10 ^ab^	125.08 ^a^	126.48 ^a^	1.19	0.03	0.006	0.417	0.469
MCH	66.45 ^c^	67.95 ^bc^	68.75 ^b^	71.70 ^a^	0.59	0.001	0.003	0.002	0.423
MCHC	58.45	55.78	55.03	56.88	0.86	0.42	0.155	0.429	0.732
Liver enzyme activities									
ALT, U/L	4.75	4.75	4.25	5.25	0.55	0.71	1.000	0.324	0.564
AST, U/L	247.50	248.75	256.25	225.00	3.30	0.77	0.871	0.323	0.812
ALK, U/L	4473	6428	4548	2924	29.53	0.58	0.937	0.253	0.461

Hematology = RBC: Red blood cells; WBC: White blood cells; H/L ratio: Heterophils per Lymphocytes ratio; MCV: Mean corpuscular volume; MCH: Mean corpuscular hemoglobin; MCHC: Mean corpuscular hemoglobin concentration; Liver enzyme activities = ALT: alanine transaminase; AST: aspartate aminotransferase; ALK: alkaline phosphatase; ^a–c^ Means within a row with different superscripts are different (*p* < 0.05); SEM: Standard error of mean; N/A: Not applicable; Contrast 1: CABP vs. CABP+F, CABP+L, CABP+FL; Contrast 2: CABP+F, CABP+L vs. CABP+FL; Contrast 3: CABP+F vs. CABP+L.

**Table 7:** Effects of experimental diets on intestinal morphology in broiler, when 24 and 35 d.

Items	Treatments				SEM	*p*-Value	Orthogonal Contrast		
	CABP	CABP+F	CABP+L	CABP+FL			Contrast 1	Contrast 2	Contrast 3
24 d									
Duodenum									
Villus height	1126.92	1347.87	864.47	1329.42	10.23	0.22	0.769	0.286	0.082
Crypt depth	222.72	253.18	200.38	199.00	3.94	0.38	0.847	0.359	0.164
V/C ratio	5.04	5.32	4.31	6.71	0.54	0.60	0.439	0.021	0.151
Jejunum									
Villus height	665.02	777.00	817.70	826.95	7.77	0.57	0.222	0.791	0.753
Crypt depth	98.60	167.97	131.75	158.17	3.85	0.23	0.090	0.763	0.290
V/C ratio	6.74 ^a^	4.65 ^c^	6.30 ^ab^	5.23 ^bc^	0.47	0.03	0.020	0.563	0.020
Ileum									
Villus height	656.77	725.73	626.02	786.67	8.87	0.75	0.685	0.462	0.561
Crypt depth	121.00	211.30	105.27	182.97	4.07	0.19	0.276	0.554	0.074
V/C ratio	5.43	3.43	6.83	4.30	0.88	0.29	0.679	0.590	0.094
35 d									
Duodenum									
Villus height	1433.95	1596.13	1699.93	1514.22	11.19	0.75	0.4536	0.570	0.700
Crypt depth	315.87	247.65	295.37	292.73	4.09	0.35	0.245	0.505	0.227
V/C ratio	4.54	6.43	5.84	5.24	0.76	0.49	0.247	0.427	0.637
Jejunum									
Villus height	1146.02	1203.55	1182.27	994.82	9.11	0.63	0.895	0.241	0.904
Crypt depth	194.10	215.28	205.15	244.18	5.29	0.83	0.978	0.312	0.855
V/C ratio	5.91	6.27	5.98	4.26	1.03	0.78	0.959	0.615	0.887
Ileum									
Villus height	953.13	898.60	1116.38	957.90	9.43	0.67	0.807	0.764	0.288
Crypt depth	327.48	217.07	204.25	212.35	5.02	0.18	0.048	0.971	0.812
V/C ratio	2.92	4.13	5.52	4.82	0.69	0.18	0.069	0.991	0.216

V/C ratio: villus height-to-crypt depth ratio; ^a–c^ Means within a row with different superscripts are different (*p* < 0.05); SEM: Standard error of means; Contrast 1: CABP vs. CABP+F, CABP+L, CABP+FL; Contrast 2: CABP+F, CABP+L vs. CABP+FL; Contrast 3: CABP+F vs. CABP+L.

## Data Availability

The data supporting the findings of this study are available within the article. Any further information can be obtained from the corresponding author upon reasonable request.
